# Supplementary material for: Interactome overlap between risk genes of epilepsy and targets of anti-epileptic drugs
Source: PLoS One. 2022 Aug 25;17(8):e0272428. doi: 10.1371/journal.pone.0272428 (PMC9409560; doi:10.1371/journal.pone.0272428)
Supplement: S1 Table — (DOCX) [file pone.0272428.s001.docx]

Supplementary Table1. The risks genes of epilepsy

| Gene Name |
| --- |
| ADSL  ALDH7A1  ALG13  ARHGEF9  ARX  ATP1A2  ATP6AP2  ATXN1  BCL11A  BRD7  C3orf33  CACNA1A  CDKL5  CHD2  CHRNA2  CHRNA4  CHRNA7  CHRNB2  CLN2  CLN3  CLN5  CLN6  CLN8  CNTNAP2  CSTB  CTSD  DEPDC5  DNAJC5  DNM1  DYRK1A  EEF1A2  EPM2A  FANCL  FOLR1  FOXG1  GABRA1  GABRA2  GABRB2  GABRB3  GABRG2  GAMT  GATM  GJA1  GNAO1  GOSR2  GRIK1  GRIN1  GRIN2A  GRIN2B  HCN1  HEATR3  HNRNPU  IQSEC2  KANSL1  KCNA2  KCNAB1  KCNB1  KCNC1  KCNJ10  KCNMA1  KCNN2  KCNQ2  KCNQ3  KCNT1  KCTD7  LGI1  MAGI2  MBD5  MECP2  MEF2C  MFSD8  NHLRC1  NR2F1  NRXN1  PCDH19  PCDH7  PIGA  PIGO  PIGV  PNKP  PNPO  POLG  PPT1  PRICKLE1  PRICKLE2  PRRT2  QARS  SCARB2  SCN1A  SCN1B  SCN2A  SCN3A  SCN8A  SCN9A  SIK1  SLC13A5  SLC25A22  SLC2A1  SLC33A1  SLC35A2  SLC6A1  SLC6A8  SLC9A6  SPTAN1  STAT4  STX1B  STXBP1  SYNGAP1  TBC1D24  TCF4  TPP1  TSC1  TSC2  TTC21B  UBE3A  WDR45  WWOX  ZEB2 |
