# Supplementary material for: Interactome overlap between risk genes of epilepsy and targets of anti-epileptic drugs
Source: PLoS One. 2022 Aug 25;17(8):e0272428. doi: 10.1371/journal.pone.0272428 (PMC9409560; doi:10.1371/journal.pone.0272428)
Supplement: S2 Table — (DOCX) [file pone.0272428.s002.docx]

Supplementary Table2 : The target genes of antiepileptic drugs

| ATC Codes | Name | Drug Bank ID | Target |
| --- | --- | --- | --- |
| N03AX24 | Cannabidiol | DB09061 | CNR1 |
| N03AX24 | Cannabidiol | DB09061 | CNR2 |
| N03AX24 | Cannabidiol | DB09061 | GPR12 |
| N03AX24 | Cannabidiol | DB09061 | GLRA1 |
| N03AX24 | Cannabidiol | DB09061 | GLRB |
| N03AX24 | Cannabidiol | DB09061 | GLRA3 |
| N03AX24 | Cannabidiol | DB09061 | GPR18 |
| N03AX24 | Cannabidiol | DB09061 | GPR55 |
| N03AX24 | Cannabidiol | DB09061 | HTR1A |
| N03AX24 | Cannabidiol | DB09061 | HTR2A |
| N03AX24 | Cannabidiol | DB09061 | CHRNA7 |
| N03AX24 | Cannabidiol | DB09061 | OPRD1 |
| N03AX24 | Cannabidiol | DB09061 | OPRM1 |
| N03AX24 | Cannabidiol | DB09061 | PPARG |
| N03AX24 | Cannabidiol | DB09061 | TRPV1 |
| N03AX24 | Cannabidiol | DB09061 | CACNA1G |
| N03AX24 | Cannabidiol | DB09061 | CACNA1H |
| N03AX24 | Cannabidiol | DB09061 | CACNA1I |
| N03AX24 | Cannabidiol | DB09061 | TRPA1 |
| N03AX24 | Cannabidiol | DB09061 | TRPM8 |
| N03AX24 | Cannabidiol | DB09061 | TRPV2 |
| N03AX24 | Cannabidiol | DB09061 | TRPV3 |
| N03AX24 | Cannabidiol | DB09061 | TRPV4 |
| N03AX24 | Cannabidiol | DB09061 | VDAC1 |
| N03AX24 | Cannabidiol | DB09061 | HTR3A |
| N03AX24 | Cannabidiol | DB09061 | ADORA1 |
| N03AX24 | Cannabidiol | DB09061 | PTGS1 |
| N03AX24 | Cannabidiol | DB09061 | PTGS2 |
| N03AX24 | Cannabidiol | DB09061 | ACAT1 |
| N03AX24 | Cannabidiol | DB09061 | CYP17A1 |
| N03AX24 | Cannabidiol | DB09061 | HMGCR |
| N03AX24 | Cannabidiol | DB09061 | GSR |
| N03AX24 | Cannabidiol | DB09061 | GPX1 |
| N03AX24 | Cannabidiol | DB09061 | IDO1 |
| N03AX24 | Cannabidiol | DB09061 | CYP1B1 |
| N03AX24 | Cannabidiol | DB09061 | NQO1 |
| N03AX24 | Cannabidiol | DB09061 | CAT |
| N03AX24 | Cannabidiol | DB09061 | CYP3A5 |
| N03AX24 | Cannabidiol | DB09061 | CYP2D6 |
| N03AX24 | Cannabidiol | DB09061 | SOD1 |
| N03AX24 | Cannabidiol | DB09061 | CYP1A2 |
| N03AX24 | Cannabidiol | DB09061 | CYP3A7 |
| ATC Codes | Name | Drug Bank ID | Target |
| N03AX24 | Cannabidiol | DB09061 | AANAT |
| N03AX24 | Cannabidiol | DB09061 | NAAA |
| N03AX23 | Brivaracetam | DB05541 | SV2A |
| N03AX23 | Brivaracetam | DB05541 | SCN1A |
| N03AX23 | Brivaracetam | DB05541 | SCN10A |
| N03AX23 | Brivaracetam | DB05541 | SCN11A |
| N03AX23 | Brivaracetam | DB05541 | SCN2A |
| N03AX23 | Brivaracetam | DB05541 | SCN3A |
| N03AX23 | Brivaracetam | DB05541 | SCN4A |
| N03AX23 | Brivaracetam | DB05541 | SCN5A |
| N03AX23 | Brivaracetam | DB05541 | SCN7A |
| N03AX23 | Brivaracetam | DB05541 | SCN8A |
| N03AX23 | Brivaracetam | DB05541 | SCN9A |
| N03AX23 | Brivaracetam | DB05541 | SCN1B |
| N03AX23 | Brivaracetam | DB05541 | SCN2B |
| N03AX23 | Brivaracetam | DB05541 | SCN3B |
| N03AX23 | Brivaracetam | DB05541 | SCN4B |
| N03AX22 | Perampanel | DB08883 | GRIA1 |
| N03AX21 | Ezogabine | DB04953 | KCNQ2 |
| N03AX21 | Ezogabine | DB04953 | KCNQ3 |
| N03AX21 | Ezogabine | DB04953 | KCNQ4 |
| N03AX21 | Ezogabine | DB04953 | KCNQ5 |
| N03AX18 | Lacosamide | DB06218 | SCN9A |
| N03AX18 | Lacosamide | DB06218 | SCN3A |
| N03AX18 | Lacosamide | DB06218 | SCN10A |
| N03AX17 | Stiripentol | DB09118 | GABRA1 |
| N03AX17 | Stiripentol | DB09118 | GABRA2 |
| N03AX17 | Stiripentol | DB09118 | GABRA3 |
| N03AX17 | Stiripentol | DB09118 | GABRA4 |
| N03AX17 | Stiripentol | DB09118 | GABRA5 |
| N03AX17 | Stiripentol | DB09118 | GABRA6 |
| N03AX17 | Stiripentol | DB09118 | GABRB1 |
| N03AX17 | Stiripentol | DB09118 | GABRB2 |
| N03AX17 | Stiripentol | DB09118 | GABRB3 |
| N03AX17 | Stiripentol | DB09118 | GABRD |
| N03AX17 | Stiripentol | DB09118 | GABRE |
| N03AX17 | Stiripentol | DB09118 | GABRG1 |
| N03AX17 | Stiripentol | DB09118 | GABRG2 |
| N03AX17 | Stiripentol | DB09118 | GABRG3 |
| N03AX17 | Stiripentol | DB09118 | GABRP |
| N03AX17 | Stiripentol | DB09118 | GABRQ |
| N03AX17 | Stiripentol | DB09118 | LDHA |
| N03AX17 | Stiripentol | DB09118 | LDHB |
| ATC Codes | Name | Drug Bank ID | Target |
| N03AX16 | Pregabalin | DB00230 | CACNA2D1 |
| N03AX15 | Zonisamide | DB00909 | SCN1A |
| N03AX15 | Zonisamide | DB00909 | SCN2A |
| N03AX15 | Zonisamide | DB00909 | SCN3A |
| N03AX15 | Zonisamide | DB00909 | SCN4A |
| N03AX15 | Zonisamide | DB00909 | SCN5A |
| N03AX15 | Zonisamide | DB00909 | SCN9A |
| N03AX15 | Zonisamide | DB00909 | SCN11A |
| N03AX15 | Zonisamide | DB00909 | SCN1B |
| N03AX15 | Zonisamide | DB00909 | SCN2B |
| N03AX15 | Zonisamide | DB00909 | SCN3B |
| N03AX15 | Zonisamide | DB00909 | SCN4B |
| N03AX15 | Zonisamide | DB00909 | CACNA1G |
| N03AX15 | Zonisamide | DB00909 | CACNA1H |
| N03AX15 | Zonisamide | DB00909 | CACNA1I |
| N03AX15 | Zonisamide | DB00909 | CA1 |
| N03AX15 | Zonisamide | DB00909 | CA2 |
| N03AX15 | Zonisamide | DB00909 | CA3 |
| N03AX15 | Zonisamide | DB00909 | CA4 |
| N03AX15 | Zonisamide | DB00909 | CA5A |
| N03AX15 | Zonisamide | DB00909 | CA5B |
| N03AX15 | Zonisamide | DB00909 | CA6 |
| N03AX15 | Zonisamide | DB00909 | CA7 |
| N03AX15 | Zonisamide | DB00909 | CA8 |
| N03AX15 | Zonisamide | DB00909 | CA9 |
| N03AX15 | Zonisamide | DB00909 | CA10 |
| N03AX15 | Zonisamide | DB00909 | CA11 |
| N03AX15 | Zonisamide | DB00909 | CA12 |
| N03AX15 | Zonisamide | DB00909 | CA13 |
| N03AX15 | Zonisamide | DB00909 | CA14 |
| N03AX15 | Zonisamide | DB00909 | MAOB |
| N03AX15 | Zonisamide | DB00909 | MAOA |
| N03AX14 | Levetiracetam | DB01202 | SV2A |
| N03AX14 | Levetiracetam | DB01202 | CACNA1B |
| N03AX12 | Gabapentin | DB00996 | CACNA2D1 |
| N03AX12 | Gabapentin | DB00996 | CACNA2D2 |
| N03AX12 | Gabapentin | DB00996 | CACNA1B |
| N03AX12 | Gabapentin | DB00996 | ADORA1 |
| N03AX12 | Gabapentin | DB00996 | KCNQ3 |
| N03AX12 | Gabapentin | DB00996 | KCNQ5 |
| N03AX11 | Topiramate | DB00273 | GABRA1 |
| N03AX11 | Topiramate | DB00273 | SCN1A |
| N03AX11 | Topiramate | DB00273 | SCN10A |
| ATC Codes | Name | Drug Bank ID | Target |
| N03AX11 | Topiramate | DB00273 | SCN11A |
| N03AX11 | Topiramate | DB00273 | SCN2A |
| N03AX11 | Topiramate | DB00273 | SCN3A |
| N03AX11 | Topiramate | DB00273 | SCN4A |
| N03AX11 | Topiramate | DB00273 | SCN5A |
| N03AX11 | Topiramate | DB00273 | SCN7A |
| N03AX11 | Topiramate | DB00273 | SCN8A |
| N03AX11 | Topiramate | DB00273 | SCN9A |
| N03AX11 | Topiramate | DB00273 | GRIK1 |
| N03AX11 | Topiramate | DB00273 | GRIK2 |
| N03AX11 | Topiramate | DB00273 | GRIK3 |
| N03AX11 | Topiramate | DB00273 | GRIK4 |
| N03AX11 | Topiramate | DB00273 | GRIK5 |
| N03AX11 | Topiramate | DB00273 | CA1 |
| N03AX11 | Topiramate | DB00273 | CA2 |
| N03AX11 | Topiramate | DB00273 | CA3 |
| N03AX11 | Topiramate | DB00273 | CA4 |
| N03AX11 | Topiramate | DB00273 | CACNA1C |
| N03AX11 | Topiramate | DB00273 | CACNA1D |
| N03AX11 | Topiramate | DB00273 | CACNA1F |
| N03AX11 | Topiramate | DB00273 | CACNA1S |
| N03AX11 | Topiramate | DB00273 | CACNB1 |
| N03AX11 | Topiramate | DB00273 | CACNB2 |
| N03AX11 | Topiramate | DB00273 | CACNB3 |
| N03AX11 | Topiramate | DB00273 | CACNB4 |
| N03AX11 | Topiramate | DB00273 | CACNA1E |
| N03AX10 | Felbamate | DB00949 | GRIN2B |
| N03AX10 | Felbamate | DB00949 | GRIN2A |
| N03AX09 | Lamotrigine | DB00555 | CACNA1E |
| N03AX09 | Lamotrigine | DB00555 | SCN1A |
| N03AX09 | Lamotrigine | DB00555 | SCN10A |
| N03AX09 | Lamotrigine | DB00555 | SCN11A |
| N03AX09 | Lamotrigine | DB00555 | SCN2A |
| N03AX09 | Lamotrigine | DB00555 | SCN3A |
| N03AX09 | Lamotrigine | DB00555 | SCN4A |
| N03AX09 | Lamotrigine | DB00555 | SCN5A |
| N03AX09 | Lamotrigine | DB00555 | SCN7A |
| N03AX09 | Lamotrigine | DB00555 | SCN8A |
| N03AX09 | Lamotrigine | DB00555 | SCN9A |
| N03AX09 | Lamotrigine | DB00555 | ADORA1 |
| N03AX09 | Lamotrigine | DB00555 | ADORA2A |
| N03AX09 | Lamotrigine | DB00555 | ADRA1A |
| N03AX09 | Lamotrigine | DB00555 | ADRA2A |
| ATC Codes | Name | Drug Bank ID | Target |
| N03AX09 | Lamotrigine | DB00555 | ADRB1 |
| N03AX09 | Lamotrigine | DB00555 | DRD1 |
| N03AX09 | Lamotrigine | DB00555 | DRD5 |
| N03AX09 | Lamotrigine | DB00555 | DRD2 |
| N03AX09 | Lamotrigine | DB00555 | GABRA1 |
| N03AX09 | Lamotrigine | DB00555 | GABRA2 |
| N03AX09 | Lamotrigine | DB00555 | GABRA3 |
| N03AX09 | Lamotrigine | DB00555 | GABRA4 |
| N03AX09 | Lamotrigine | DB00555 | GABRA5 |
| N03AX09 | Lamotrigine | DB00555 | GABRA6 |
| N03AX09 | Lamotrigine | DB00555 | GABRB1 |
| N03AX09 | Lamotrigine | DB00555 | GABRB2 |
| N03AX09 | Lamotrigine | DB00555 | GABRB3 |
| N03AX09 | Lamotrigine | DB00555 | GABRD |
| N03AX09 | Lamotrigine | DB00555 | GABRE |
| N03AX09 | Lamotrigine | DB00555 | GABRG1 |
| N03AX09 | Lamotrigine | DB00555 | GABRG2 |
| N03AX09 | Lamotrigine | DB00555 | GABRG3 |
| N03AX09 | Lamotrigine | DB00555 | GABRP |
| N03AX09 | Lamotrigine | DB00555 | GABRQ |
| N03AX09 | Lamotrigine | DB00555 | GABRA1 |
| N03AX09 | Lamotrigine | DB00555 | GABRA2 |
| N03AX09 | Lamotrigine | DB00555 | GABRA3 |
| N03AX09 | Lamotrigine | DB00555 | GABRA5 |
| N03AX09 | Lamotrigine | DB00555 | GABRG1 |
| N03AX09 | Lamotrigine | DB00555 | GABRG2 |
| N03AX09 | Lamotrigine | DB00555 | GABRG3 |
| N03AX09 | Lamotrigine | DB00555 | HRH1 |
| N03AX09 | Lamotrigine | DB00555 | OPRK1 |
| N03AX09 | Lamotrigine | DB00555 | CHRNA1 |
| N03AX09 | Lamotrigine | DB00555 | HTR2A |
| N03AX09 | Lamotrigine | DB00555 | HTR3A |
| N03AX09 | Lamotrigine | DB00555 | GRIA1 |
| N03AX07 | Phenacemide | DB01121 | SCN1A |
| N03AX03 | Sulthiame | DB08329 | CA2 |
| N03AG06 | Tiagabine | DB00906 | SLC6A1 |
| N03AG05 | Progabide | DB00837 | GABBR1 |
| N03AG05 | Progabide | DB00837 | GABBA1 |
| N03AG04 | Vigabatrin | DB01080 | ABAT |
| N03AG02 | Valpromide | DB04165 | limA |
| N03AG01 | Valproic acid | DB00313 | ACADSB |
| N03AG01 | Valproic acid | DB00313 | HDAC9 |
| N03AG01 | Valproic acid | DB00313 | OGDH |
| ATC Codes | Name | Drug Bank ID | Target |
| N03AG01 | Valproic acid | DB00313 | ALDH5A1 |
| N03AG01 | Valproic acid | DB00313 | SCN1A |
| N03AG01 | Valproic acid | DB00313 | SCN10A |
| N03AG01 | Valproic acid | DB00313 | SCN11A |
| N03AG01 | Valproic acid | DB00313 | SCN2A |
| N03AG01 | Valproic acid | DB00313 | SCN3A |
| N03AG01 | Valproic acid | DB00313 | SCN4A |
| N03AG01 | Valproic acid | DB00313 | SCN5A |
| N03AG01 | Valproic acid | DB00313 | SCN7A |
| N03AG01 | Valproic acid | DB00313 | SCN8A |
| N03AG01 | Valproic acid | DB00313 | SCN9A |
| N03AG01 | Valproic acid | DB00313 | SCN1B |
| N03AG01 | Valproic acid | DB00313 | SCN2B |
| N03AG01 | Valproic acid | DB00313 | SCN3B |
| N03AG01 | Valproic acid | DB00313 | SCN4B |
| N03AG01 | Valproic acid | DB00313 | HDAC2 |
| N03AG01 | Valproic acid | DB00313 | PPARA |
| N03AG01 | Valproic acid | DB00313 | PPARD |
| N03AG01 | Valproic acid | DB00313 | PPARG |
| N03AF04 | Eslicarbazepine | DB14575 | P2RX4 |
| N03AF03 | Rufinamide | DB06201 | GRM5 |
| N03AF03 | Rufinamide | DB06201 | SCN9A |
| N03AF02 | Oxcarbazepine | DB00776 | SCN1A |
| N03AF02 | Oxcarbazepine | DB00776 | SCN10A |
| N03AF02 | Oxcarbazepine | DB00776 | SCN11A |
| N03AF02 | Oxcarbazepine | DB00776 | SCN2A |
| N03AF02 | Oxcarbazepine | DB00776 | SCN3A |
| N03AF02 | Oxcarbazepine | DB00776 | SCN4A |
| N03AF02 | Oxcarbazepine | DB00776 | SCN5A |
| N03AF02 | Oxcarbazepine | DB00776 | SCN7A |
| N03AF02 | Oxcarbazepine | DB00776 | SCN8A |
| N03AF02 | Oxcarbazepine | DB00776 | SCN9A |
| N03AF02 | Oxcarbazepine | DB00776 | SCN1B |
| N03AF02 | Oxcarbazepine | DB00776 | SCN2B |
| N03AF02 | Oxcarbazepine | DB00776 | SCN3B |
| N03AF02 | Oxcarbazepine | DB00776 | SCN4B |
| N03AF01 | Carbamazepine | DB00564 | SCN1A |
| N03AF01 | Carbamazepine | DB00564 | SCN10A |
| N03AF01 | Carbamazepine | DB00564 | SCN11A |
| N03AF01 | Carbamazepine | DB00564 | SCN2A |
| N03AF01 | Carbamazepine | DB00564 | SCN3A |
| N03AF01 | Carbamazepine | DB00564 | SCN4A |
| N03AF01 | Carbamazepine | DB00564 | SCN5A |
| ATC Codes | Name | Drug Bank ID | Target |
| N03AF01 | Carbamazepine | DB00564 | SCN7A |
| N03AF01 | Carbamazepine | DB00564 | SCN8A |
| N03AF01 | Carbamazepine | DB00564 | SCN9A |
| N03AF01 | Carbamazepine | DB00564 | CHRNA4 |
| N03AF01 | Carbamazepine | DB00564 | NR1I2 |
| N03AE01 | Clonazepam | DB01068 | GABRA1 |
| N03AE01 | Clonazepam | DB01068 | GABRA2 |
| N03AE01 | Clonazepam | DB01068 | GABRA3 |
| N03AE01 | Clonazepam | DB01068 | GABRA4 |
| N03AE01 | Clonazepam | DB01068 | GABRA5 |
| N03AE01 | Clonazepam | DB01068 | GABRA6 |
| N03AE01 | Clonazepam | DB01068 | GABRB1 |
| N03AE01 | Clonazepam | DB01068 | GABRB2 |
| N03AE01 | Clonazepam | DB01068 | GABRB3 |
| N03AE01 | Clonazepam | DB01068 | GABRD |
| N03AE01 | Clonazepam | DB01068 | GABRE |
| N03AE01 | Clonazepam | DB01068 | GABRG1 |
| N03AE01 | Clonazepam | DB01068 | GABRG2 |
| N03AE01 | Clonazepam | DB01068 | GABRG3 |
| N03AE01 | Clonazepam | DB01068 | GABRP |
| N03AE01 | Clonazepam | DB01068 | GABRQ |
| N03AE01 | Clonazepam | DB01068 | GABRA1 |
| N03AE01 | Clonazepam | DB01068 | GABRA2 |
| N03AE01 | Clonazepam | DB01068 | GABRA3 |
| N03AE01 | Clonazepam | DB01068 | GABRA5 |
| N03AE01 | Clonazepam | DB01068 | GABRG1 |
| N03AE01 | Clonazepam | DB01068 | GABRG2 |
| N03AE01 | Clonazepam | DB01068 | GABRG3 |
| N03AE01 | Clonazepam | DB01068 | NR1I2 |
| N03AD51 | Ethosuximide | DB00593 | CACNA1G |
| N03AD03 | Methsuximide | DB05246 | CACNA1G |
| N03AD01 | Ethosuximide | DB00593 | CACNA1G |
| N03AC02 | Trimethadione | DB00347 | CACNA1G |
| N03AC01 | Paramethadione | DB00617 | CACNA1I |
| N03AB54 | Mephenytoin | DB00532 | SCN5A |
| N03AB54 | Mephenytoin | DB00532 | NR1I2 |
| N03AB52 | Phenytoin | DB00252 | SCN5A |
| N03AB52 | Phenytoin | DB00252 | SCN1A |
| N03AB52 | Phenytoin | DB00252 | NR1I2 |
| N03AB52 | Phenytoin | DB00252 | SCN1B |
| N03AB52 | Phenytoin | DB00252 | SCN1A |
| N03AB52 | Phenytoin | DB00252 | KCNH2 |
| N03AB52 | Phenytoin | DB00252 | CACNA1C |
| ATC Codes | Name | Drug Bank ID | Target |
| N03AB52 | Phenytoin | DB00252 | CACNA1D |
| N03AB52 | Phenytoin | DB00252 | CACNA1F |
| N03AB52 | Phenytoin | DB00252 | CACNA1S |
| N03AB52 | Phenytoin | DB00252 | CACNB1 |
| N03AB52 | Phenytoin | DB00252 | CACNB2 |
| N03AB52 | Phenytoin | DB00252 | CACNB3 |
| N03AB52 | Phenytoin | DB00252 | CACNB4 |
| N03AB52 | Phenytoin | DB00252 | CACNA1A |
| N03AB52 | Phenytoin | DB00252 | SCN2A |
| N03AB52 | Phenytoin | DB00252 | SCN8A |
| N03AB52 | Phenytoin | DB00252 | GABRA1 |
| N03AB52 | Phenytoin | DB00252 | GABRA2 |
| N03AB52 | Phenytoin | DB00252 | GABRA3 |
| N03AB52 | Phenytoin | DB00252 | GABRA4 |
| N03AB52 | Phenytoin | DB00252 | GABRA5 |
| N03AB52 | Phenytoin | DB00252 | GABRA6 |
| N03AB52 | Phenytoin | DB00252 | GABRB1 |
| N03AB52 | Phenytoin | DB00252 | GABRB2 |
| N03AB52 | Phenytoin | DB00252 | GABRB3 |
| N03AB52 | Phenytoin | DB00252 | GABRD |
| N03AB52 | Phenytoin | DB00252 | GABRE |
| N03AB52 | Phenytoin | DB00252 | GABRG1 |
| N03AB52 | Phenytoin | DB00252 | GABRG2 |
| N03AB52 | Phenytoin | DB00252 | GABRG3 |
| N03AB52 | Phenytoin | DB00252 | GABRP |
| N03AB52 | Phenytoin | DB00252 | GABRQ |
| N03AB05 | Fosphenytoin | DB01320 | SCN5A |
| N03AB04 | Mephenytoin | DB00532 | SCN5A |
| N03AB04 | Mephenytoin | DB00532 | NR1I2 |
| N03AB02 | Phenytoin | DB00252 | SCN5A |
| N03AB02 | Phenytoin | DB00252 | SCN1A |
| N03AB02 | Phenytoin | DB00252 | NR1I2 |
| N03AB02 | Phenytoin | DB00252 | SCN1B |
| N03AB02 | Phenytoin | DB00252 | SCN3A |
| N03AB02 | Phenytoin | DB00252 | KCNH2 |
| N03AB02 | Phenytoin | DB00252 | CACNA1C |
| N03AB02 | Phenytoin | DB00252 | CACNA1D |
| N03AB02 | Phenytoin | DB00252 | CACNA1F |
| N03AB02 | Phenytoin | DB00252 | CACNA1S |
| N03AB02 | Phenytoin | DB00252 | CACNB1 |
| N03AB02 | Phenytoin | DB00252 | CACNB2 |
| N03AB02 | Phenytoin | DB00252 | CACNB3 |
| N03AB02 | Phenytoin | DB00252 | CACNB4 |
| ATC Codes | Name | Drug Bank ID | Target |
| N03AB02 | Phenytoin | DB00252 | CACNA1A |
| N03AB02 | Phenytoin | DB00252 | SCN2A |
| N03AB02 | Phenytoin | DB00252 | SCN8A |
| N03AB02 | Phenytoin | DB00252 | GABRA1 |
| N03AB02 | Phenytoin | DB00252 | GABRA2 |
| N03AB02 | Phenytoin | DB00252 | GABRA3 |
| N03AB02 | Phenytoin | DB00252 | GABRA4 |
| N03AB02 | Phenytoin | DB00252 | GABRA5 |
| N03AB02 | Phenytoin | DB00252 | GABRA6 |
| N03AB02 | Phenytoin | DB00252 | GABRB1 |
| N03AB02 | Phenytoin | DB00252 | GABRB2 |
| N03AB02 | Phenytoin | DB00252 | GABRB3 |
| N03AB02 | Phenytoin | DB00252 | GABRD |
| N03AB02 | Phenytoin | DB00252 | GABRE |
| N03AB02 | Phenytoin | DB00252 | GABRG1 |
| N03AB02 | Phenytoin | DB00252 | GABRG2 |
| N03AB02 | Phenytoin | DB00252 | GABRG3 |
| N03AB02 | Phenytoin | DB00252 | GABRP |
| N03AB02 | Phenytoin | DB00252 | GABRQ |
| N03AB01 | Ethotoin | DB00754 | SCN5A |
| N03AB01 | Ethotoin | DB00754 | NR1I2 |
| N03AA30 | Metharbital | DB00463 | GABRA1 |
| N03AA30 | Metharbital | DB00463 | GABRA2 |
| N03AA30 | Metharbital | DB00463 | GABRA3 |
| N03AA30 | Metharbital | DB00463 | GABRA4 |
| N03AA30 | Metharbital | DB00463 | GABRA5 |
| N03AA30 | Metharbital | DB00463 | GABRA6 |
| N03AA30 | Metharbital | DB00463 | CHRNA4 |
| N03AA30 | Metharbital | DB00463 | CHRNA7 |
| N03AA30 | Metharbital | DB00463 | GRIA2 |
| N03AA30 | Metharbital | DB00463 | GRIK2 |
| N03AA30 | Metharbital | DB00463 | GABRA1 |
| N03AA30 | Metharbital | DB00463 | GABRA2 |
| N03AA30 | Metharbital | DB00463 | GABRA3 |
| N03AA30 | Metharbital | DB00463 | GABRA4 |
| N03AA30 | Metharbital | DB00463 | GABRA5 |
| N03AA30 | Metharbital | DB00463 | GABRA6 |
| N03AA30 | Metharbital | DB00463 | GABRB1 |
| N03AA30 | Metharbital | DB00463 | GABRB2 |
| N03AA30 | Metharbital | DB00463 | GABRB3 |
| N03AA30 | Metharbital | DB00463 | GABRD |
| N03AA30 | Metharbital | DB00463 | GABRE |
| N03AA30 | Metharbital | DB00463 | GABRG1 |
| ATC Codes | Name | Drug Bank ID | Target |
| N03AA30 | Metharbital | DB00463 | GABRG2 |
| N03AA30 | Metharbital | DB00463 | GABRG3 |
| N03AA30 | Metharbital | DB00463 | GABRP |
| N03AA30 | Metharbital | DB00463 | GABRQ |
| N03AA03 | Primidone | DB00794 | GABRA1 |
| N03AA03 | Primidone | DB00794 | GABRA2 |
| N03AA03 | Primidone | DB00794 | GABRA3 |
| N03AA03 | Primidone | DB00794 | GABRA4 |
| N03AA03 | Primidone | DB00794 | GABRA5 |
| N03AA03 | Primidone | DB00794 | GABRA6 |
| N03AA03 | Primidone | DB00794 | CHRNA4 |
| N03AA03 | Primidone | DB00794 | CHRNA7 |
| N03AA03 | Primidone | DB00794 | GRIA2 |
| N03AA03 | Primidone | DB00794 | GRIK2 |
| N03AA03 | Primidone | DB00794 | GABRA1 |
| N03AA03 | Primidone | DB00794 | GABRA2 |
| N03AA03 | Primidone | DB00794 | GABRA3 |
| N03AA03 | Primidone | DB00794 | GABRA4 |
| N03AA03 | Primidone | DB00794 | GABRA5 |
| N03AA03 | Primidone | DB00794 | GABRA6 |
| N03AA03 | Primidone | DB00794 | GABRB1 |
| N03AA03 | Primidone | DB00794 | GABRB2 |
| N03AA03 | Primidone | DB00794 | GABRB3 |
| N03AA03 | Primidone | DB00794 | GABRD |
| N03AA03 | Primidone | DB00794 | GABRE |
| N03AA03 | Primidone | DB00794 | GABRG1 |
| N03AA03 | Primidone | DB00794 | GABRG2 |
| N03AA03 | Primidone | DB00794 | GABRG3 |
| N03AA03 | Primidone | DB00794 | GABRP |
| N03AA03 | Primidone | DB00794 | GABRQ |
| N03AA02 | Phenobarbital | DB01174 | GABRA1 |
| N03AA02 | Phenobarbital | DB01174 | CHRNA4 |
| N03AA02 | Phenobarbital | DB01174 | CHRNA7 |
| N03AA02 | Phenobarbital | DB01174 | GRIA2 |
| N03AA02 | Phenobarbital | DB01174 | GRIK2 |
| N03AA02 | Phenobarbital | DB01174 | GRIN1 |
| N03AA02 | Phenobarbital | DB01174 | GRIN2A |
| N03AA02 | Phenobarbital | DB01174 | GRIN2B |
| N03AA02 | Phenobarbital | DB01174 | GRIN2C |
| N03AA02 | Phenobarbital | DB01174 | GRIN2D |
| N03AA02 | Phenobarbital | DB01174 | GRIN3A |
| N03AA02 | Phenobarbital | DB01174 | GRIN3B |
| N03AA02 | Phenobarbital | DB01174 | NR1I2 |
| ATC Codes | Name | Drug Bank ID | Target |
| N03AA01 | Methylphenobarbital | DB00849 | GABRA1 |
| N03AA01 | Methylphenobarbital | DB00849 | GABRA2 |
| N03AA01 | Methylphenobarbital | DB00849 | GABRA3 |
| N03AA01 | Methylphenobarbital | DB00849 | GABRA4 |
| N03AA01 | Methylphenobarbital | DB00849 | GABRA5 |
| N03AA01 | Methylphenobarbital | DB00849 | GABRA6 |
| N03AA01 | Methylphenobarbital | DB00849 | CHRNA4 |
| N03AA01 | Methylphenobarbital | DB00849 | CHRNA7 |
| N03AA01 | Methylphenobarbital | DB00849 | GRIA2 |
| N03AA01 | Methylphenobarbital | DB00849 | GRIK2 |
| N03AA01 | Methylphenobarbital | DB00849 | NR1I2 |
