# Supplementary material for: Interactome overlap between risk genes of epilepsy and targets of anti-epileptic drugs
Source: PLoS One. 2022 Aug 25;17(8):e0272428. doi: 10.1371/journal.pone.0272428 (PMC9409560; doi:10.1371/journal.pone.0272428)
Supplement: S3 Table — (DOCX) [file pone.0272428.s003.docx]

Supplementary Table 3. Genes of co-expression network of epilepsy

| Genes of co-expression network of epilepsy |
| --- |
| MAP3K9, ARHGAP44, ADAM22, GABRA3, RWDD2A, SYT13, GABRA1, ARNTL2, PHF23, FSTL4, CDK14, CAMK2B, SLC2A3, GUCY1B3, FAR2, SNAP91, TRO, SYT1, REEP1, KCNAB2, ATP1B3, GNB5, CDC42, CRMP1, PANX2, IPCEF1, RASAL2, UBE2T, PAK3, AMPH, RBFOX1, IMPG2, EPB41L3, KIF3C, TTC39A, SNX10, NRCAM, SMPX, SLC17A6, SLC7A8, UPRT, CDC7, SEZ6L, NEFH, VTI1B, CPNE6, EEF1A2, PAK7, MCF2, FGF14, FGF9, AAGAB, AP3B2, NIPAL2, STMN2, NCALD, NEFM, LIN7B, GPI, ATP1A3, MAST1, TFPT, RAB3A, PBX4, TSPAN13, LIMK1, DNM1, ELAVL2, APBA1, SH3GL2, MAPK8, CNTNAP1, SLC16A6, UNC119, GABRA4, INPP4B, FBXW7, ELMOD1, PPFIBP1, KCNA1, ENO2, NT5DC3, CD83, CAP2, GPLD1, PDE10A, GABRG2, FGF12, IFT57, ATP6V1A, ADAM23, POLE4, GLS, EHBP1, SLC25A12, EPHA4, PHTF1, SIPA1L2, B4GALT6, ELOVL4, CNNM1, MTRF1, SLITRK3, ODF2L, LRRC39, KIAA1549, CIT, CKS2, SEPT6, PANK2, NAPB, PCSK2, PLEKHG3, TMEM35, TUBA4A, YWHAH, FOXP2, CCDC136, MYO1B, KCNC1, FGF13, DPP6, SULT4A1, CALY, REEP2, SNAP25, SYT4, ATP8A2, MTUS2, GPR12, RTN3, MRPS36, CCNB1, VAV3, TIMM17A, BIVM, MRAP2, B4GALNT1, ACVR1B, CKAP2, TBC1D4, STXBP1, GABBR2, TUBB2A, GGH, ARHGAP20, SLC44A5, CYP2C8, ARL3, PRKG2, GABARAPL1, PRICKLE1, CLSTN3, MDGA2, RTN1, RDH12, RAB15, KCNH5, CBLN2, SOD1, XPR1, SCN1A, ZNF385B, CPNE9, CNTN4, OSBPL10, TAGLN3, NCEH1, RNF175, CDH18, GABRB2, FAXC, RSPO3, NLGN4X, NDUFB11, PRPS1, FAM135B, POLE3, INA, GAS2, PAK1, ALDOA, FAM57B, CNTN5, GPR158, CACNA1C, SLC2A13, CENPJ, GUCY1A2, PFKM, PANK1, RAB3C, NRSN1, ANAPC1, CLGN, RBMS1, GDPD1, THY1, ABCA10, UCHL1, ATP5G3, RAB6B, CNTNAP5, FAM126B, AFF2, TSPAN7, NPTN, NMNAT2, PALM2-AKAP2, GPRASP2, SV2A, ZNF233, CALM3, CHRNB2, RUSC1, KCNJ3, VSNL1, SLC16A14, SGPP2, CADPS, CDS1, SLC9B2, AGGF1, UQCRQ, RELL2, OXR1, COX6C, PDP1, DIRAS2, ZMAT4, MAMDC2, MOAP1, CACNB2, KCNC2, GABRB3, C11orf74, CCDC68, CACNG2, BSCL2, NSG1, CPLX1, FAM110B, REPS2, YWHAG, DENND5B, LONRF2, SDR16C5, KIAA0232, HS6ST2, PRKCE, MAP6, GAP43, GPR22, RASGRP1, SLC2A14, USMG5, LINGO2, NRIP3, CLTB, DIRAS1, GRIN1, SCN4B, ZDHHC22, KCNA2, NAP1L5, CPNE7, PITPNB, CHRM2, ZNF804B, CACNB4, TTC3, GALNT9, PSMG1, OPCML, TUBA8, UQCR10, KCNQ3, SLIT3, MAP7D2, CEND1, NELL2, INPP5J, WBSCR17, SV2B, AHNAK2, MORF4L1, DLEU7, NAP1L3, CYP4X1, NAP1L2, GNG2, TSPYL4, HAPLN4, GABRD, PAQR9, IL1RAP, ESRRG, SPTSSB, SCN8A, ANXA6, ADGRA1, RAD54B, MYO5A, FAM49A, RP11-566K11.2, PEG3, GFPT1, SH3BGRL2, TOX, L1CAM, GPRASP1, CHML, FAM216A, SYT3, MLLT11, KRT222, SOGA3, FXYD7, ZBED9, PLCXD2 |
