# Supplementary material for: Interactome overlap between risk genes of epilepsy and targets of anti-epileptic drugs
Source: PLoS One. 2022 Aug 25;17(8):e0272428. doi: 10.1371/journal.pone.0272428 (PMC9409560; doi:10.1371/journal.pone.0272428)
Supplement: S4 Table — (DOCX) [file pone.0272428.s004.docx]

Supplementary Table 4. Brain cell-types enrichment analysis of interaction network of risk gene of epilepsy and antiepileptic drug targets

| CellType | P | Bonferroni-P | fold_change | sd_from_mean |
| --- | --- | --- | --- | --- |
| interneurons | 0 | 0 | 1.62 | 7.82 |
| Medium Spiny Neuron | 0 | 0 | 2.27 | 9.07 |
| pyramidal CA1 | 0 | 0 | 1.49 | 6.18 |
| pyramidal SS | 0 | 0 | 1.47 | 6.04 |
| Striatal Interneuron | 5.23E-03 | 0.13 | 1.36 | 3.07 |
| astrocytes_ependymal | 0.14 | 1 | 1.18 | 1.12 |
| Hypothalamic Dopaminergic Neurons | 0.19 | 1 | 1.08 | 0.83 |
| Dopaminergic Adult | 0.23 | 1 | 1.06 | 0.69 |
| Hypothalamic GABAergic Neurons | 0.54 | 1 | 0.98 | -0.25 |
| Hypothalamic Glutamatergic Neurons | 0.62 | 1 | 0.95 | -0.44 |
| Vascular Leptomeningeal Cells | 0.84 | 1 | 0.75 | -1 |
| Oxytocin and Vasopressin Expressing Neurons | 0.96 | 1 | 0.85 | -1.59 |
| Serotonergic Neuron | 0.98 | 1 | 0.82 | -1.81 |
| Oligodendrocytes | 0.99 | 1 | 0.71 | -1.86 |
| microglia | 0.99 | 1 | 0.53 | -2.07 |
| Embryonic GABAergic Neuron | 1 | 1 | 0.81 | -2.44 |
| endothelial-mural | 1 | 1 | 0.58 | -2.33 |
| Dopaminergic Neuroblast | 1 | 1 | 0.61 | -2.93 |
| Oligodendrocyte Precursor | 1 | 1 | 0.59 | -2.91 |
| Embryonic Dopaminergic Neuron | 1 | 1 | 0.61 | -3.15 |
| Embryonic midbrain nucleus neurons | 1 | 1 | 0.65 | -3.55 |
| Neural Progenitors | 1 | 1 | 0.39 | -4.5 |
| Neuroblasts | 1 | 1 | 0.57 | -3.67 |
| Radial glia like cells | 1 | 1 | 0.53 | -4.13 |
