# Supplementary material for: Interactome overlap between risk genes of epilepsy and targets of anti-epileptic drugs
Source: PLoS One. 2022 Aug 25;17(8):e0272428. doi: 10.1371/journal.pone.0272428 (PMC9409560; doi:10.1371/journal.pone.0272428)
Supplement: Attachment — Submitted filename: reponse.docx [file pone.0272428.s006.docx]

**Response Letter**

**Dear editor and reviewers:**

**Thank you for your reviewing! We have revised our manuscript according to your suggestion, and our response was as follows:**

Comments to the Author

1. Is the manuscript technically sound, and do the data support the conclusions?

The manuscript must describe a technically sound piece of scientific research with data that supports the conclusions. Experiments must have been conducted rigorously, with appropriate controls, replication, and sample sizes. The conclusions must be drawn appropriately based on the data presented.

Reviewer #1: Yes

2. Has the statistical analysis been performed appropriately and rigorously?

Reviewer #1: N/A

3. Have the authors made all data underlying the findings in their manuscript fully available?

The PLOS Data policy requires authors to make all data underlying the findings described in their manuscript fully available without restriction, with rare exception (please refer to the Data Availability Statement in the manuscript PDF file). The data should be provided as part of the manuscript or its supporting information, or deposited to a public repository. For example, in addition to summary statistics, the data points behind means, medians and variance measures should be available. If there are restrictions on publicly sharing data—e.g. participant privacy or use of data from a third party—those must be specified.

Reviewer #1: No

**Response: We have added all data underlying the findings in supporting information (Supplementary Table 1-4) and data availability statement.**

4. Is the manuscript presented in an intelligible fashion and written in standard English?

PLOS ONE does not copyedit accepted manuscripts, so the language in submitted articles must be clear, correct, and unambiguous. Any typographical or grammatical errors should be corrected at revision, so please note any specific errors here.

Reviewer #1: No

**Response: We have checked our manuscript and revised them according to your suggestion.**

5. Review Comments to the Author

Please use the space provided to explain your answers to the questions above. You may also include additional comments for the author, including concerns about dual publication, research ethics, or publication ethics. (Please upload your review as an attachment if it exceeds 20,000 characters)

Reviewer #1: The authors present an interesting study systematically investigating the

potential interaction of epilepsy risk genes and antiepileptic drug targets. Although the area and novelty of the study approach have the potential for publication, I have some major and minor concerns which are listed below:

Major Concerns:

1) Please briefly explain the process of study with a graphical abstract or a flowchart showing the pipe lines of your marker selections.

**Response: We have added a flowchart of the pipelines according to your suggestions as Figure 1.**

2) Did you used any ethical analysis due to genetic risk factors? How you exclude the normal variations in each population? Any Haplogroup analysis were conducted?

**Response: We obtained risk genes of epilepsy identified by both common variants and rare variants, of which common variants are extracted from the largest trans-ethnic meta-analyses of genome-wide association studies currently[12], which included 15,212 individuals with epilepsy and 29,677 controls and identified 16 genome-wide significant loci with P-value<5.0×10-8. Rare variants were identified by exome-sequencing under the largest sample size of 1165 cases and 3877 controls [13], as the article demonstrated, these variants in normal population have been investigated in the article and demonstrated that they are exceptionally rare in the general population.**

3) What is the rationale behind choosing each data bae? Please briefly explain it for each data base.

**Response:**

**1.DisGeNET database[14] and MalaCards database[15] are comprehensive databases with disease associated genes and variants, which provided evidence of risk genes.**

**2.DrugBank[16] is a web-based database containing comprehensive molecular information about drugs, their mechanisms of action, interactions, and their targets, it provided drug targets information with literature supported evidence.**

**3.Corum[17], BioPlex[18], CCSB[19], Integral[20] and BioGRID[21] are protein-protein interaction databases that are commonly used in network analysis, to comprehensively capture the PPI data, we integrated five PPI database together.**

**4.KI dataset [28-30] is a superset of brain scRNA-seq data from the Karolinska Institutet (KI) including 24 cell types from brain regions of neocortex, hippocampus, hypothalamus, striatum and midbrain, which has been used to identify enrichment of risk genes in major brain disorders [11,27].**

4) It seems that false discovery rate (FDR) < 0.01 used for multiple correction. While the Bonferroni multiple correction test is more conservative why you didn’t use Bonferroni? Is that possible that some of those markers excluded if you use more conservative tests?

**Response: For Expression Weighted Cell type Enrichment (EWCE), we have revised the multiple correction method to Bonferroni multiple correction test, under this threshold, genes were significantly enriched in four brain cell types (interneurons, Medium Spiny Neuron, CA1 pyramidal Neuron, Somatosensory pyramidal Neuron), and we revised the results in “6.** **Brain cell-type enrichment analysis of** **interaction network between antiepileptic drug targets and risk genes of epilepsy”, Fig 3 and Supplementary Table 4. For pathway enrichment analysis, FDR was the most commonly used method, if Bonferroni multiple correction test was used, meaningful pathways might be excluded.**

5) I was wondering that why you didn’t used the WGCNA R software package to analysis of weighted gene co-expression network analysis (WGCNA)? Please explain the reason in manuscript or use the analysis and add the results in your revised manuscript.

**Response: Our study was based on interactome data, which was not expression data. However, according to your valuable suggestion, we used a co-expression network of 320 genes (M30) related with epilepsy, which was obtained by gene co-expression network analysis (WGCNA [24] and DiffCoEx[25]) in the brain reported by a previous study[26]. We identified 22 target genes (*CACNA1A,CHRNA4,CHRNA7,GABRA1,GABRA2,GABRB2,GABRG2,GRIK1,GRIN1,GRIN2B,KCNQ2,KCNQ3,SCN1A,SCN2A, SCN3A,SCN8A* and *SCN9A*) were overlapped with risk genes of epilepsy.. Fisher’s Exact Test demonstrated a significant over-representation of targets of antiepileptic drug in the risk genes of epilepsy (odds ratio [OR] =11.80, P=1.30 ×10 −15). We have revised them in related Methods and Results section.**

6) Discussion part should explain the related genetic markers much more mechanistically to help the readers understand why future treatments may us these data to choose more targeted drug compounds.

**Response: we have add a paragraph in “Discussion” in the manuscript as:**

**“*Moreover, when we compared genes in this PPI network with genes from a co-expression network of epilepsy [26] (Supplementary Table 3), we also identified a significant enrichment of genes in our PPI network in the co-expression network of epilepsy, with 22 gene overlapped, interestingly, all the 22 genes were antiepileptic drug targets, in which 7 genes were also risk genes (GABRA1, GABRB2, GABRG2, GRIN1, KCNQ3, SCN1A, and SCN8A). Voltage-gated sodium channels (VGSCs) play a critical role in generation of action potentials, SCN1A, SCN2A, SCN3A, SCN8A and SCN1B have been identified to be associated with a spectrum of epilepsy phenotypes and neurodevelopmental disorders[40]. Besides, So far, the most widespread viewpoint considered that GABAA receptor, as an isomer receptor that binds to GABA, affects the excitability of nerve cells by stimulating chloride ions influx into the postsynaptic membrane and exerts antiepileptic effect[41]. It was also reported a personalized therapy in a GRIN1 mutated girl with intellectual disability and epilepsy [42]. Our results demonstrated that it was important to know the functional effect (Loss-of-function versus Gain-of-function) of a variant for genes which were both risk genes and drug targets to orient therapeutic decisions.*”**

7) Please add a paragraph explaining the limitations of the study.

**Response: we have add a paragraph before “Conclusion” in the manuscript as:**

**“*In our study, we explored the network interaction between antiepileptic drug and risk genes of epilepsy by systematic data collection and integrative analysis. However, there were still some inevitable limitations. First, although we used interactome data by integrating five comprehensive PPI databases, there might still exist interactions between risk genes and drug targets that could not be identified by current interactome data, which are worthy of being explored with the update of interctome data. Second, since the number of cells taken in the single-cell data sets accounts for only a small portion of the whole brain tissue, they may not represent all types of brain cells and need further validation. Third, all the results were obtained by systems biology and network analyses based on data from public databases, which might only reveal underlying mechanisms with currently existing information and need further experimental validation.*”**

Minor concerns:

1(Use abbreviations correctly in whole manuscript.

**Response: We have checked our manuscript and revised them according to your suggestion.**

2) There are other minor issues (such as diction, editing, and linguistic errors) that need to be corrected in the revised version of the MS. It is highly recommended that a native English speaker edits the entire manuscript (particularly the introduction section) before submission of the revised MS.

**Response:We have checked our manuscript and revised them according to your suggestion.**

3) In line 113, please indicate the version of Cytoscape software that you used.

**Response: Cytoscape Version 3.8.2 was used.**
